# The Anatomy of the SARS-CoV-2 Biomedical Literature: Introducing the CovidX Network Algorithm for Drug Repurposing Recommendation

**DOI:** 10.2196/21169

**Published:** 2020-08-20

**Authors:** Lyndsey Elaine Gates, Ahmed Abdeen Hamed

**Affiliations:** 1 School of Nursing Norwich University Northfield, VT United States; 2 School of Cybersecurity, Data Science, and Computing Norwich University Northfield, VT United States

**Keywords:** health, informatics, COVID-19 treatment, drug repurposing, network algorithm, ranking, drug, biomedical, antiviral, COVID-19

## Abstract

**Background:**

Driven by the COVID-19 pandemic and the dire need to discover an antiviral drug, we explored the landscape of the SARS-CoV-2 biomedical publications to identify potential treatments.

**Objective:**

The aims of this study are to identify off-label drugs that may have benefits for the coronavirus disease pandemic, present a novel ranking algorithm called CovidX to recommend existing drugs for potential repurposing, and validate the literature-based outcome with drug knowledge available in clinical trials.

**Methods:**

To achieve such objectives, we applied natural language processing techniques to identify drugs and linked entities (eg, disease, gene, protein, chemical compounds). When such entities are linked, they form a map that can be further explored using network science tools. The CovidX algorithm was based upon a notion that we called “diversity.” A diversity score for a given drug was calculated by measuring how “diverse” a drug is calculated using various biological entities (regardless of the cardinality of actual instances in each category). The algorithm validates the ranking and awards those drugs that are currently being investigated in open clinical trials. The rationale behind the open clinical trial is to provide a validating mechanism of the PubMed results. This ensures providing up to date evidence of the fast development of this disease.

**Results:**

From the analyzed biomedical literature, the algorithm identified 30 possible drug candidates for repurposing, ranked them accordingly, and validated the ranking outcomes against evidence from clinical trials. The top 10 candidates according to our algorithm are hydroxychloroquine, azithromycin, chloroquine, ritonavir, losartan, remdesivir, favipiravir, methylprednisolone, rapamycin, and tilorone dihydrochloride.

**Conclusions:**

The ranking shows both consistency and promise in identifying drugs that can be repurposed. We believe, however, the full treatment to be a multifaceted, adjuvant approach where multiple drugs may need to be taken at the same time.

## Introduction

### Background

In December 2019, the first known cases of the coronavirus disease (COVID-19), caused by the severe acute respiratory syndrome coronavirus 2 (SARS-CoV-2), were identified in humans in Wuhan, China. Transmission was thought to have occurred through contact with animals at a wet market, although the exact animal-to-human transmission incidence is still unknown at the time of this publication [1]. Since then, SARS-CoV-2 infections have been reported worldwide, with the greatest numbers of infections in the United States, Russia, and Europe [2]. With no immunity among humans, at the time of this writing, SARS-CoV-2 has infected over 4.5 million individuals and killed over 300,000 individuals worldwide with the potential for many more fatalities without a known cure or vaccination [3].

With high mortality rates thus far resulting from the SARS-CoV-2 infection, true morbidity rates are still unknown. Studies have shown that cardiac injury occurred in up to 28% of patients hospitalized with a SARS-CoV-2 infection [4], which can increase the risk of death later on. The systemic inflammatory response that occurs in severe SARS-CoV-2 infection may result in hypoxemia and increased cardiac demand on an already taxed cardiovascular system. Previous research has shown that viral respiratory infections may increase the risk of myocardial infarctions in patients, prompting some researchers to focus on infection prevention initiatives as strategies for lowering risk of cardiovascular disease [4].

Due to the uncertainty of the processes involved in SARS-CoV-2 infection and treatment, novel approaches are needed. One approach is drug repurposing, where drugs that are approved for other diseases are investigated to determine whether they are safe and effective for different conditions. Drug repurposing efforts have resulted in a great number of treatment options for disorders such as Sildenafil treatment for erectile dysfunction (previously intended for hypertension and angina), Itraconazole for lung and prostate cancer treatment (previously used only for antifungal purposes), and Saracatinib therapy for investigation into Alzheimer disease reversal (previously thought to be a failure drug for anticancer purposes) [5].

The purpose of this study is to identify drugs in circulation that could be repurposed to offer potential benefits of use in treating patients infected with SARS-CoV-2.

### Computational Methods for Drug Repurposing

The idea of investigating drugs that are already designed for a given indication to serve a new purpose is not new to science [6]. This idea has become recently more prominent due to the human advancement in computational sciences and the possibilities of processing large data sets on a high-performance computing unit [7]. Drug repurposing can provide effective therapies, such as in the case of invasive fungal infections [8]. Computational repurposing offers the following two approaches: (1) target-based repurposing, where a drug is interacting with a gene or a protein, and (2) disease-based, where a drug is found connected to new indications [9].

Over the past decades, various approaches have been proposed using machine learning, network science, and text mining [10]. Cheng et al [11] introduced a bipartite network-based approach for a drug-target inference method that was proven useful for detecting drug-target interactions in the molecular polypharmacological domain. Wu et al [12] presented a heterogeneous network clustering approach for associating drugs with disease. The nodes of the network presented nodes as drugs and diseases, while edges represented a shared gene or biological process, etc [12].

Text mining played a significant role in advancing biomedical informatics in general [13] and computational drug research in particular [14,15]. Wu et al [16] developed computational models to retrieve drug-drug interaction and drug-gene interaction evidence from PubMed abstracts.

Andronis et al [17] presented a review on the application of literature mining using semantic ontology and controlled vocabularies as tools to explore the drug repurposing research. Loging et al [18] presented literature databases such as Medline as knowledge-driven systems to identify novel uses for drugs that span the therapeutic pipeline. Deftereos et al [19] demonstrated the importance of both text mining and networks to discover new drug uses and adverse drug reactions. The purpose of text mining is used to extract the names of the genes, disease, and biological processes. Networks presented the associations among the names to allow the discovery of the molecular mechanisms underlying disease and links to existing drugs that may be used [19]. Nabirotchkin et al [20] presented a combined networks and text mining approach to advance the multi-keyword search for drug repurposing (eg, two drugs and one target).

As search capabilities advanced, ranking algorithms also emerged. Luo et al [21] presented a ranking algorithm to produce a drug repurposing recommendation system. Karatszas et al [22] presented a web-based tool, which they called composite drug reranking scoring, to identify the most promising drugs and chemical substances to test. Hamed et al [23] combined both literature text mining and networks to rank existing drugs based on the biological specificity of a drug to explore their similarity and whether they can be repurposed. Similarly, we present a new ranking algorithm that is based on a new notion, which they called *variability*. The methods section describes the details of each component of this research.

## Methods

### Overview

**Figure 1:**
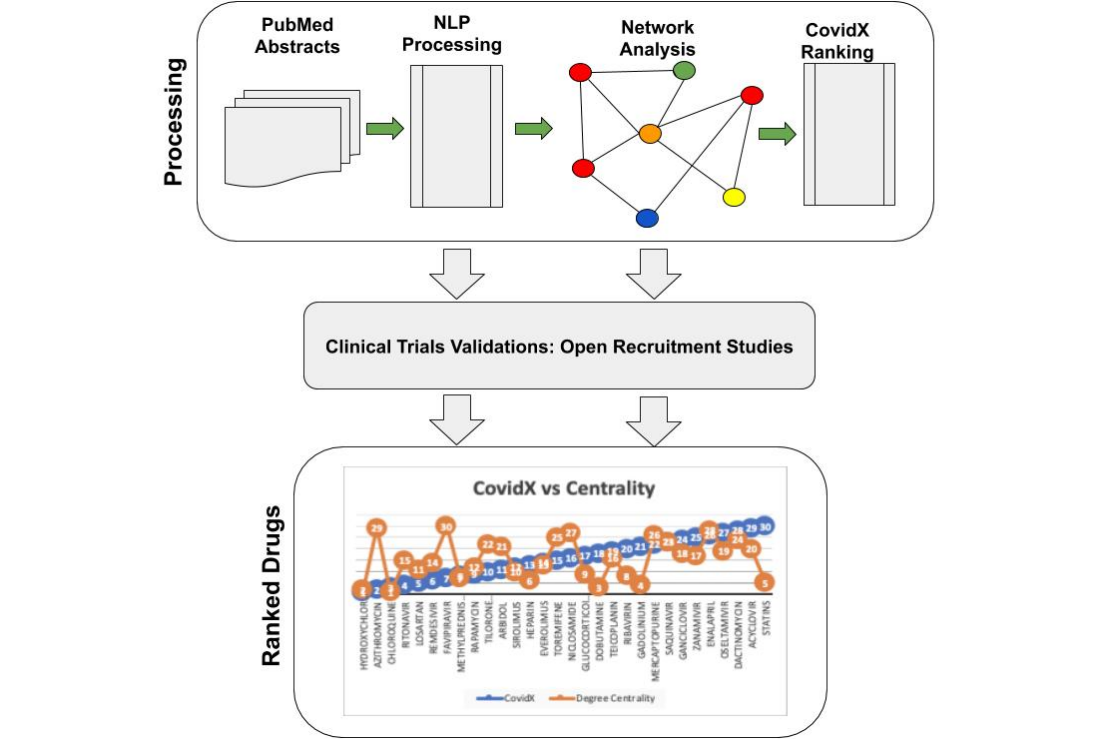
Workflow diagram showing how the processing of PubMed abstracts using NLP produces a network of drugs (symbolized in red) and various biological features (in various colors). The workflow shows the clinical trials validation steps before it produces the final ranking results of the CovidX algorithm. NLP: natural language processing.

The method that we used in this exploratory study is described as follows: named entity recognition [24], drug-entities network construction, and drug scoring using an algorithm that we titled CovidX. Before the algorithm produced the final ranking, it factored in whether a drug was under current investigation in clinical trials. The algorithm awarded those drugs by a given weight based on the frequency of studies that were open for recruitment. Each step is presented along with the data set used in the analysis onward. Figure 1 shows the data workflow and the various steps of data processing and making drug repurposing recommendations.

### Data Sets

Starting from the PubMed web portal, we searched for “SARS-CoV-2” keywords. This search process produced 941 publications captured in the abstracts and metadata. Since the focus of this work is a computational study about mining literature, the focus of the data was the text embedded in the abstracts. The study kept track of each article ID, known as PMID, and the abstract text. The abstracts were the data grounds for text analysis, and the PMID ensured that the extracted entities of the same article were linked together. Another complementary data set that we extracted from the clinical trials web portal included those studies related to COVID-19 and that were also recruiting participants. This data set was comprised of 433 records, which were analyzed for their title, conditions, and intervention sections for COVID-19, and the coexisting drugs in the PubMed data set.

### Instruments and Experiment’s Environment

The data preparing, analysis, experiment, and implementation used here were done entirely using Python Jupyter Notebook [25] to facilitate the reproducibility of this study. We used specific packages to do the work: TextBlob [26] for text processing, NetworkX [27] for network analysis and algorithm implementation, and the Python version iGraph [28] for network visualization.

### Entity Recognition and Network Construction

In the first step, we applied known natural language techniques [29] to identify and extract the entities mentioned in the biomedical abstracts. Named entity recognition [24] provides the means of identifying and extracting entities such as drug names, genes, proteins, cell lines, organism, and chemical compounds. Some of these tools are ontology-driven and identify entities if they exist in biological ontology [30]. Biological ontology, such as the Gene Ontology [31-33], offer great tools since they have been manually vetted by domain experts. They also play a significant role in the identification of the biological entities of this paper. All identified names are qualified by their ontology (GO: membrane, CHEBI: chloroquine) or feature type (disease: obstructive coronary disease).

**Figure 2:**
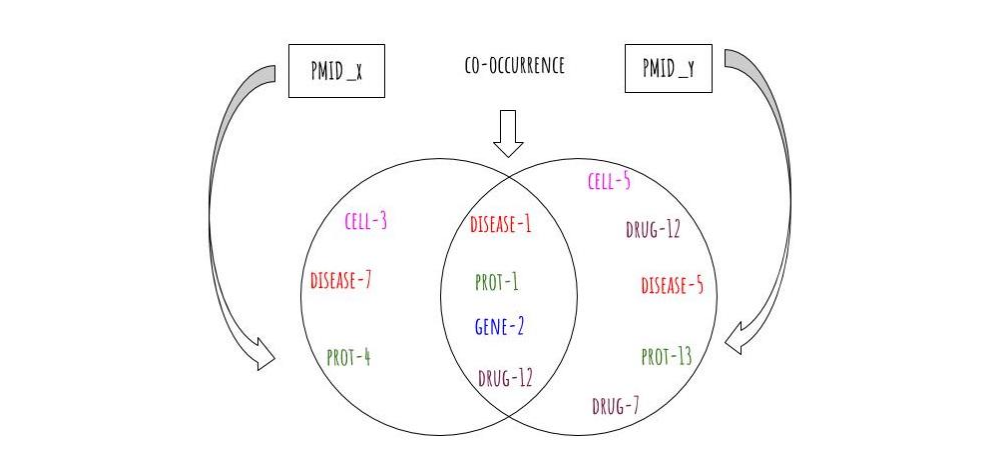
Two hypothetical PubMed Abstracts (PMIDx and PMIDy). Each abstract contributes some biological entities. The entities in the intersection namely (disease-1, prot-1, gene-2, and drug-12) contribute to a network of co-occurring entities that become the grounds for further exploration. In the presence of a larger data set of abstracts and more biological features, the network becomes an interesting ground for exploration and potential discoveries.

In the second step, we constructed a (drug-entity) map using the biological entities that have been identified in the first step. The map is constructed using a heuristic: “if a set of targets belongs to a given abstract, and co-occurring with a drug, then they must be related. How they are related is demonstrated in the scope of this work.” This offers a connectivity mechanism between the drugs and the various targets. As new drugs are extracted and connected with their corresponding targets, the map grows naturally. Figure 2 demonstrates the extraction of various feature types and links with its original PMID.

**Figure 3:**
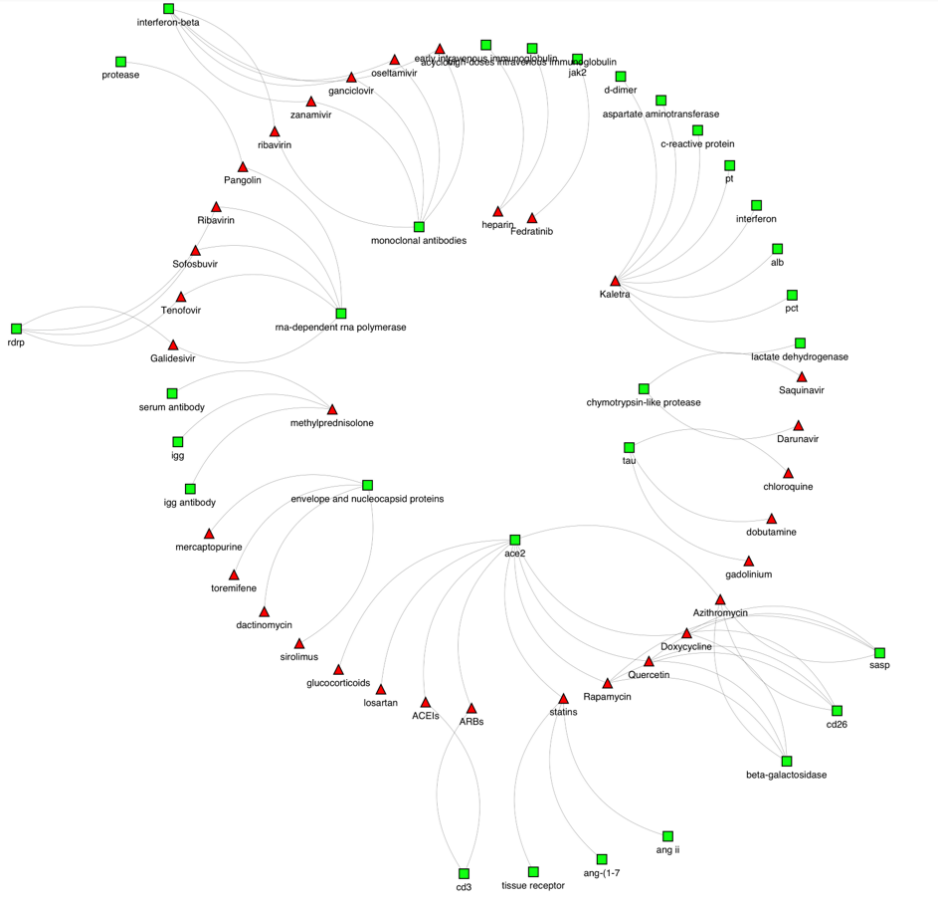
The connections between drugs and protein targets. The drugs nodes are represented as red triangles and the protein nodes are represented by green squares. Here we note that various drugs have targeted one specific protein (ACE2), while other drugs have been found linked to more than one protein as in the case with methylprednisolone. ACEIs: angiotensin-converting enzyme inhibitors; ace2: angiotensin-converting enzyme 2; ang: angiotensin; ANG II: angiotensin II; ARBs: angiotensin II receptor blockers; igg: immunoglobulin G; jak2: Janus kinase 2; RdRp: RNA-dependent RNA polymerase.

This provides us with an interesting landscape in terms of structure and connectivity for exploration and discovery. The network naturally grows when a drug, for example, in one abstract is mentioned in another abstract. This is the case when a drug target is addressed in various publications by the same or different authors. The more the abstracts have features in common, the more the network grows in links and structure. Here we represent the drug-entity map as a graph, G=(V; E), where (V) represents the set of drugs on one hand and the features on the other hand as nodes, and (E) denotes the connections from drugs to the entities co-occurring in the data set. Figure 3 presents drug-protein as a subset network of the entire network of drug-feature extracted in this process.

### CovidX Ranking Algorithm

The CovidX ranking algorithm we present here is based on a simple yet powerful heuristics: the more knowledge known about the drug, the higher the likelihood that it can be repurposed. The following criteria were used to determine whether the knowledge could be considered relevant:

- The diversity of links to the named entities identified using the natural language processing framework used earlier (eg, gene, protein, cell line, chemical, disease)
- Whether the drug is currently being investigated in an ongoing clinical trial [34] specialized in COVID-19

The notion of diversity is quantified by the degree of drug to each unique feature type. Diversity is captured by a binary matrix where a value of 1 represents a connection and 0 represents no connection to the category. For instance, if a drug is found linked to a given protein instance, this registers a value of 1. On the other hand, if a drug is not linked to a gene, this places a value of a 0 in the corresponding column. In the case where a drug is found linked to various instances of the same category (eg, a drug is linked to three different proteins), this still contributes a value of 1 not 3.

The scoring mechanism is based on two contributing factors: (1) the log of the diversity score for each drug mentioned in the PubMed data set and (2) the normalized frequency of each drug in the clinical trial data set [34]. The justification for this heuristic is that a drug can be promising but has not been investigated in a clinical trial yet. On the other hand, it is not the case that every clinical trial results in a successful outcome. Some trials may fail in late phases.

The given weight of these two factors addresses both the issues of promise or failure. The following are the algorithmic steps necessary to produce a ranking score:

1. Generate an incident matrix for all drugs in the network
2. Convert the incident matrix into a diversity matrix, one that is based on the node category (eg, drug), not the actual label (eg, chloroquine)
3. For each category a drug is linked with, place a value of 1; otherwise, place a value of 0
4. Calculate the column variability score: log of the value for total number of categories divided by the sum of the 1 values
5. If a drug is active in a clinical trial, add the drug name and its frequency score to the list of potential candidates
6. Rank drugs in an ascending order based on the variability score, the lower the value the higher the rank

#### Algorithm Formalism

Suppose N denotes a matrix of drugs and their corresponding neighbors. The first column G is a set of drugs instances {g1, g2, g3, g4}, while the remaining number of columns denote neighboring nodes of each drug in C. Each instance of G has a set of biological neighbors of type (genes, proteins, cell type, cell line). This can be mathematically represented with a matrix where the first column is the drug instances. Each row is linked to the actual instances of feature type. For simplification, equation 1 purposes only instances of four different feature types {w, x, y, z}.

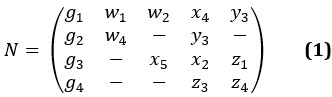



Here we note that each feature in the A matrix corresponds to a feature type with an index. This alphabetical symbol represents the type, and the index represents a unique instance of that feature. For example, drug g1 has two instances of type w,

namely, w1 and w2. Similarly, drug g4 has two instances of type z, namely, z3 and z4. This A matrix can be transformed into a new binary matrix, which we call the “diversity” matrix denoted as D. Table 1 shows a concrete representation of each drug and the expression of the diversity notion. Due to the page size limitation, we included only the most significant five features, namely, cell line, cell type, disease, DNA, and protein.

This matrix is described with the following set of columns: {g1, g2, g3, g4}. Here the rows are reserved for the feature types (ie, {w, x, y, z}), which we introduced earlier. For each drug linked to a specific instance (of a feature type), a value of 1 is placed in the corresponding row; otherwise, a value of 0 is recorded. Equation 2 shows the newly transformed matrix D.

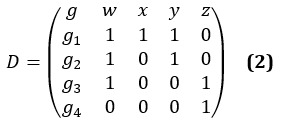



To summarize the D matrix and produce a rank for each drug, we calculated its transpose R according to the following equation R=*D^T^*. Each rank is derived from the sum of each *g_i_* column. Equation 3 shows the new matrix R and the sum of each column. For better readability, we also list the column and row labels.

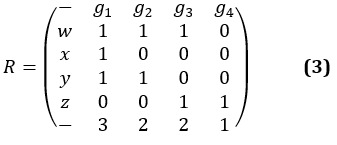



Using the column sums calculated, we ranked the drugs according to equation 4. It is to be noted that s is the column sum and n is the total number of features. This formula is inspired by the inverse term frequency56. The second part of the equation takes into consideration the frequency weight of each drug studied in clinical trials by dividing the frequencies q by the total number of clinical trials m. For those drugs that were not studied in clinical trials, we assigned a random weight in the range of zero and the lowest frequency of drugs.

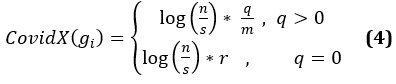



**Figure 4:**
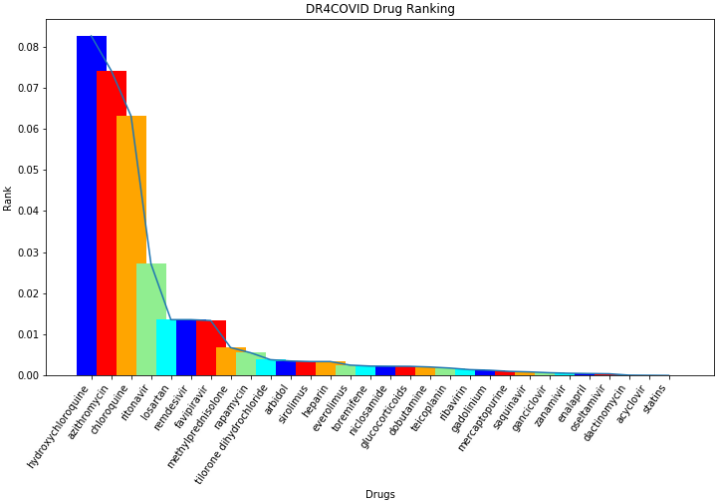
The drugs that are found in the literature and the derived ranking using the CovidX algorithm. The algorithm takes into consideration the diversity notion introduced earlier and the frequency of a drug that is currently being studied in a clinical trial. The combination of these two factors has produced a ranking mechanism to enable the selection of candidate drugs for repurposing. The diagram shows the top-10 listed according to the significance.

Figure 4 shows the final ranking results produced by our algorithm. It is striking to see that hydroxychloroquine is ranked at the top on the list despite the various news article about the side effects. We believe this is because it is the most investigated in clinical trial.

**Table 1:** Summary of the binary matrix of drugs-entity resulted from the natural language procressing.^a^

Drug	Cell line	Cell type	Disease	DNA	Protein
Hydroxychloroquine	1	1	1	0	1
Chloroquine	1	0	1	1	1
Sirolimus	1	0	1	0	1
Rapamycin	0	0	1	0	1
Everolimus	0	0	1	0	1
Methylprednisolone	0	0	1	0	1
Statins	0	0	1	0	1
Ribavirin	0	1	1	1	1
Dobutamine	0	0	1	1	1
Gadolinium	0	0	1	1	1
Tilorone dihydrochloride	0	0	1	0	0
Enalapril	0	0	1	0	1
Losartan	0	0	1	1	1
Glucocorticoids	0	0	1	1	1
Saquinavir	0	0	1	0	1
Heparin	1	0	1	1	1
Azithromycin	0	0	1	1	0
Dactinomycin	1	0	1	0	0
Toremifene	1	0	1	0	0
Mercaptopurine	1	0	1	0	0
Remdesivir	0	0	1	1	1
Ritonavir	0	1	1	1	0
Teicoplanin	0	1	1	1	1
Arbidol	0	0	1	0	1
Zanamivir	0	0	1	0	1
Ganciclovir	0	0	1	0	1
Oseltamivir	0	0	1	0	1
Acyclovir	0	0	1	0	1
Favipiravir	0	0	1	0	1
Niclosamide	0	0	1	0	1

^a^The table is a representation of the diversity matrix. When a drug is found connected to a feature type, it scores a value of 1 regardless of how many instances of the feature. If no instances are found, then a value of 0 is registered in the corresponding type of the given feature.

## Results

### Pathophysiology of SARS-CoV-2

It is speculated that SARS-CoV-2 enters the body by binding to the angiotensin-converting enzyme 2 (ACE2) coreceptors in a host cell, which are found in tissues of the lung, heart, kidney, brain, and gastrointestinal system. The actual ACE2 enzyme is involved with the renin-angiotensin-aldosterone system (RAAS), where it breaks down angiotensin II (ANG II) and generates angiotensin 1-7, thereby decreasing blood pressure in the body [35]. There is also speculation that SARS-CoV-2 enters the body through the cluster of differences (CD)26 receptor, as this is the host receptor for Middle East respiratory syndrome–related coronavirus (MERS-CoV) [36].

Current research is focused on determining the exact pathogenesis of SARS-CoV-2. One study from China showed that in patients hospitalized with a SARS-CoV-2 infection, common disease complications included acute respiratory distress syndrome (ARDS), acute cardiac injury, and secondary infection [37]. Clinical features of the disease thus far have shown to be an initial increase in the secretion of interleukin (IL)–4 and IL-10, which are T helper (Th)–2 cytokines and suppress inflammation ([37] as cited in [38]), and a Th-1 cell hyper-response that is thought to lead to the ARDS associated with severe acute respiratory syndrome (SARS; [39] as cited in [38]).

In less symptomatic patients, symptoms may involve gastrointestinal dysfunction or no symptoms at all. In those who are more highly symptomatic, early symptoms include fever, cough, nasal congestion, and fatigue. These symptoms have been reported to progress to dyspnea and pneumonia, which can be confirmed with computed tomography (CT) imaging. The sequelae of SARS-CoV-2 pneumonia includes decreased oxygen saturation, changes in blood gas composition, ground glass opacities on CT imaging, and other alveolar abnormalities. Laboratory markers include lymphopenia, elevated C-reactive protein, and elevated levels of proinflammatory cytokines [40]. Some research has shown significant lower T cell and B cell levels; natural killer levels; and CD3, CD4, and CD8 lymphocyte levels [41]. Liver dysfunction has been reported with abnormal levels of alanine aminotransferase and aspartate aminotransferase [42]. Procalcitonin levels have shown to be normal in patients admitted to the hospital for SARS-CoV-2 infection and then increased in those admitted to intensive care units [3]. Higher plasma levels of lactate dehydrogenase have also been reported [43].

Additionally, some patients with a SARS-CoV-2 infection have progressed to even more severe respiratory distress, sepsis, and septic shock [44]. Related laboratory markers have included an elevated D-dimer and elevated fibrinogen levels [45]. Some cases have shown complications related to coagulopathy and disseminated intravascular coagulation (DIC) [46].

Nucleocapsids are structures that consist of a protein that encloses a piece of RNA. With a virus, the nucleocapsid can be targeted by drugs that can open this type of envelope for access and eventual modification or destruction.

### Cardiovascular Drugs

Due to the viral binding of ACE2 receptors, concerns have been raised regarding the use of antihypertensive and cholesterol-lowering agents in patients. For example, the use of ACE inhibitors and ANG II receptor blockers, in addition to statin medications, have been shown to enhance the effects of ACE2 and, therefore, the risk of a SARS-CoV-2 infection. On the other hand, discontinuing RAAS blockade has the potential to increase the cardiovascular and respiratory complications that result from SARS-CoV-2 infection related to increased blood pressure, inflammation, and fibrosis [35].

### Anticoagulant Drugs

Patients on anticoagulant therapy and particularly those on vitamin K antagonists (VKA) have shown worse clinical outcomes when hospitalized with SARS-CoV-2 infection related to the variability in metabolic processes during the diseased state. Direct oral anticoagulant therapy is often discouraged due to potential drug interactions with proposed antiviral and other therapy. As a result, it has been suggested for patients to be switched to low-molecular weight heparin or unfractionated heparin to avoid complications of variable VKA therapy. In those patients for whom VKA therapy is required, there should be close monitoring of prothrombin time and international normalized ratio [47].

Additionally, for prophylactic treatment of coagulopathy and DIC, and for the prevention of clots related to long-term bed rest of patients being treated for a SARS-CoV-2 infection, heparin therapy has been proposed; however, the effective dosing regimen has yet to be determined [46].

### Drugs With Antiviral Activity

Outbreaks of coronavirus, prior to that of SARS-CoV-2, have largely been treated with chemical agents that target enzymes involved with DNA replication and synthesis (helicase and polymerase) and the protein catalysis needed for functional viruses (protease), as well as agents that modify the immune response, such as interferon and corticosteroids ([48], as cited in [49]). In particular, drugs that target chymotrypsin-like protease have been attractive due to its significance in the viral replication process [50].

SARS-CoV-2 is 97% genetically identical to SARS infections seen in the past. The RNA-dependent RNA polymerase (RdRp) involved with SARS-CoV-2 has become a target for several drugs used to treat hepatitis, HIV, and the Ebola virus. One modeling study suggested that drugs such as ribavirin, remdesivir, sofosbuvir, galidesivir, and tenovir, which tightly bind to RdRp, could be effective in treating SARS-CoV-2 infections [51].

Recent studies have shown agents that typically interfere with protease, such as lopinavir, ritonavir, and saquinavir, may interact with the main protease involved in SARS-CoV-2 [49]. Darunavir is a more potent protease inhibitor and currently used for HIV infection in conjunction with ritonavir, an antiretroviral agent [52].

Arbidol, a broad-spectrum antiviral medication used to treat influenza, has been shown in an in vitro study to inhibit SARS-CoV-2 infection [53]. However, no conclusive efficacy of arbidol in human use has been reported to date. Oseltamivir, a neuramidase inhibitor and another medication used to treat influenza, has not shown any effectiveness in the prevention or treatment of SARS-CoV-2 infection [54]. Similarly, zanamivir, another neuramidase inhibitor, has not demonstrated efficacy against SARS-related coronavirus (SARS-CoV) infections in the past [55].

Several medications have been hypothesized to have a reduction in the senescence that occurs with SARS-CoV-2 infection. Rapamycin, a protein synthesis inhibitor, when combined with azithromycin, has shown promise with preventing the onset of senescence and inhibiting viral replication. Doxycycline, which is an antibiotic medication, has shown antiviral activity in mammals, as well as a reduction of IL-6 serum levels and an antisenescent effect. Quercetin, which is a pigment found in food and plants, and found in supplement form, has been proposed as a potential binder of SARS-CoV-2 to reduce virus-host interactions with ACE2 receptors [36].

Tilorone is a broad-spectrum antiviral medication that has shown promise for MERS-CoV, Chikungunya, Ebola, and Marburg infections. It has been shown to induce interferon [56]. Teicoplanin, which is a glycopeptide antibiotic drug, has shown to be effective against SARS-CoV, Ebola, influenza, hepatitis C, HIV, and MERS-CoV in vitro [57].

Acyclovir and ganciclovir are nucleoside analog antiviral drugs. They are typically used to target DNA viruses such as herpes simplex virus and varicella-zoster viruses but have not shown efficacy against older SARS-CoV infections [55].

### Antimalarial Drugs

Hydroxychloroquine is a drug that is currently used for malarial infection, rheumatoid arthritis (RA), and systemic lupus erythematosus (SLE). Its anti-inflammatory and immunomodulatory mechanisms of action in RA and SLE are largely unknown, and its antimalarial activity is thought to be related to inhibiting the polymerization of heme molecules within parasites [58]. Additionally, chloroquine has been shown to prevent the induction and accumulation of beta-galactosidase, which is a biomarker of cellular senescence [36].

Both in vitro studies and physiologically based pharmacokinetic models have recently shown that both chloroquine and hydroxychloroquine have inhibited the growth of SARS-CoV-2, with hydroxychloroquine being more potent and with a greater safety profile than chloroquine. The mechanism is unknown but thought to be related to the immunomodulatory mechanisms found inherent to these drugs [59].

An open-label, nonrandomized clinical trial from France showed a significant effect of hydroxychloroquine and an even greater drug potency when it was combined with azithromycin, an antibiotic medication [54].

### Corticosteroid Drugs

Methylprednisolone is a steroid medication largely used for anti-inflammatory purposes in endocrine disorders, rheumatic disorders, collagen diseases, dermatologic disease, allergic states, ophthalmic diseases, respiratory diseases, hematologic disorders, neoplastic diseases, edematous states, gastrointestinal diseases, nervous system disorders, and others [60]. Methylprednisolone, used in conjunction with tocilizumab and lopinavir therapy, has shown promise in the treatment of SARS-CoV-2 infection through decreased oxygen requirements, decrease in C-reactive protein, increased lymphocyte levels, decreased fever, and improved chest tightness, potentially related to depression of inflammatory cytokine involvement [61].

### Convalescent Plasma Therapy

Patients who have contracted SARS-CoV-2 infection have shown to seroconvert for immunoglobulin G and immunoglobulin M antibodies, which may serve to be useful for diagnosis of either infection or exposure [62]. Current research is being conducted on the administration of antibodies to patients with SARS-CoV-2 infection with the intent of neutralizing the virus. Options for this treatment include monoclonal antibodies, synthetic preparations grown in animal hosts, or administration of antibodies harvested from patients who have recovered from SARS-CoV-2 infection. This therapy has shown efficacy in past treatment of SARS coronavirus (SARS-CoV-1) and Middle East respiratory syndrome infections [63].

### Other Therapy

The use of type 1 interferons including interferon alpha and interferon beta is being investigated for treatment of SARS-CoV-2. This therapy is typically used for treatment of multiple sclerosis or other disorders impacted by immunomodulatory drugs. However, multiple in vitro and in vivo studies have been conducted on the effect of interferons on MERS-CoV and SARS-CoV with either inconclusive results or a mild improvement of symptoms [64].

One drug used for myeloproliferative neoplasms, Fedratinib, targets the receptors for Janus kinase 2, which is a gene involved with the growth and division of cells. There is some speculation that this could be used for modulation of the inflammatory processes involved in a SARS-CoV-2 infection [65].

Some researchers have suggested using serum albumin as a delivery vehicle for potential drugs to ensure their uptake [66].

## Discussion

### Principal Findings

Here we discuss the results of the CovidX algorithm and how such results are compared against counterparts produced using common centrality methods (eg, the degree and eigenvector centrality). The research identified 30 drugs found in the SARS-CoV-2 literature abstracts publicly available in PubMed. According to CovidX, each drug is ranked based on two factors: (1) the variability factor of the known information about each drug and (2) whether the drug is currently under investigation in ClinicalTrials.gov [34]. The following is a list of the top 10 according to the algorithm: hydroxychloroquine, azithromycin, chloroquine, ritonavir, losartan, remdesivir, favipiravir, methylprednisolone, rapamycin, and tilorone dihydrochloride. Given the top 10 ranking generated, it is promising to see what the algorithm produced and highlighted. It is a well-known fact that drugs such as hydroxychloroquine, chloroquine, and remdesivir are occupying the headlines of major news networks and websites. It is a valid argument to claim that the frequency of the drug being investigated in a clinical trial has impacted the ranking mechanism. In fact, we believe the contribution of the clinical trial factor has certainly provided a measurable notion of support and guided the algorithm to stay within an acceptable range of being promising. The clinical trials alone, however, masked the importance of other drugs that have been studied in publications but not in clinical trials.

**Figure 5:**
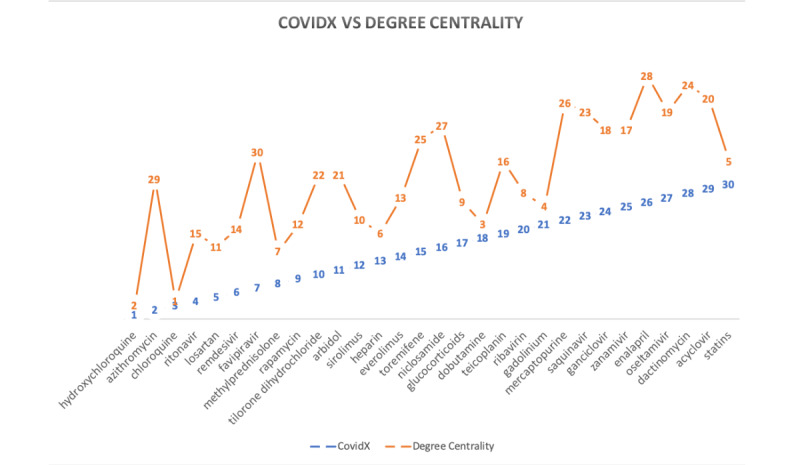
How the degree of centrality as a ranking mechanism is compared to the CovidX algorithm. The horizontal axis is the drugs listed in alphabetical order, and the vertical axis shows the ranking.

To better our understanding of the CovidX rankings, we compared the result of the algorithm against closely related centrality measures (more specifically, the degree of centrality, which studies the drug and the number of connected neighbors regardless of their type, and the eigenvector centrality, which is comparable to the sum of the columns that CovidX’s variability measure is based upon). Table 2 shows the list of drugs and their degree centrality and eigenvector centrality. Comparing the results of produced ranks using the two measures have provided a common overlapping theme. Both measures show that only three drugs among the top 10 were found in common with the ones produced by CovidX, namely, hydroxychloroquine, chloroquine, and methylprednisolone. Figure 5 shows how the CovidX ranking is compared to the degree centrality of the network.

**Table 2:** Summary of the network centrality analysis.

Drug	Degree centrality	Eigenvector centrality
Acyclovir	0.363636	0.069286
Arbidol	0.189394	0.030948
Azithromycin	0.151515	0.002197
Chloroquine	0.151515	0.496042
Dactinomycin	0.121212	0.015459
Dobutamine	0.098485	0.256032
Enalapril	0.090909	0.0101
Everolimus	0.090909	0.034237
Favipiravir	0.083333	0.014957
Gadolinium	0.068182	0.256032
Ganciclovir	0.060606	0.069286
Glucocorticoids	0.05303	0.030512
Heparin	0.05303	0.09582
Hydroxychloroquine	0.037879	0.182739
Losartan	0.037879	0.014163
Mercaptopurine	0.037879	0.015459
Methylprednisolone	0.037879	0.118721
Niclosamide	0.037879	0.021294
Oseltamivir	0.037879	0.069286
Rapamycin	0.037879	0.034237
Remdesivir	0.030303	0.063039
Ribavirin	0.022727	0.153115
Ritonavir	0.015152	0.063039
Saquinavir	0.015152	0.016058
Sirolimus	0.015152	0.049697
Statins	0.015152	0.05124
Teicoplanin	0.015152	0.063039
Tilorone dihydrochloride	0.007576	0.017456
Toremifene	0.007576	0.015459
Zanamivir	0.007576	0.069286

Our algorithm shows that hydroxychloroquine is our top candidate, but it was scored as second in the degree of centrality. It is surprising to see that azithromycin scored second according to the CovidX algorithm, but it was scored 29th according to the degree. We also notice some close terms in the rank of chloroquine (third vs first) and methylprednisolone (eighth vs seventh).

**Figure 6:**
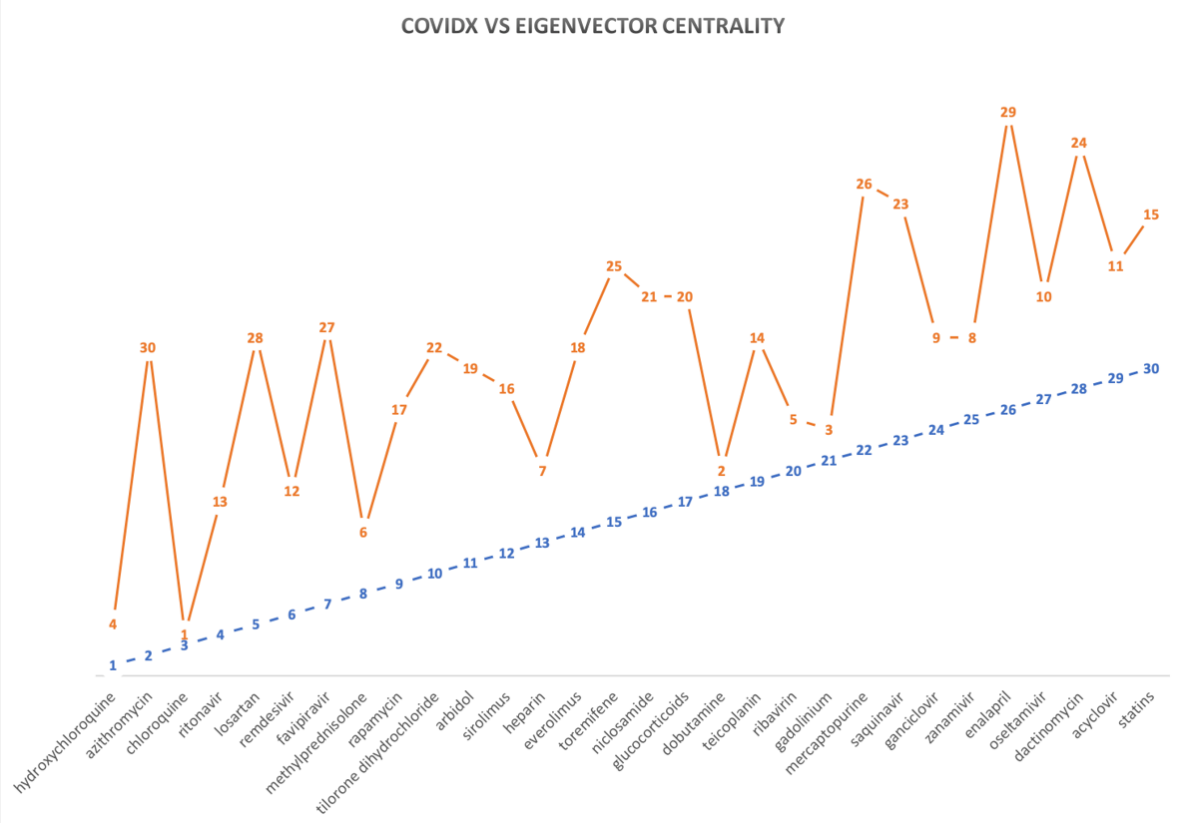
A comparison of the ranks computed by the CovidX algorithm vs the eigenvector centrality of each drug. The horizontal axis is the drugs listed in alphabetical order, and the vertical axis shows the ranking.

We also noted that other promising drugs such as remdesivir scored 14th in the degree and 12th in the eigenvector centrality, while it scored sixth according to CovidX. By observing the lower-ranked drugs produced by CovidX, we also noticed that statin drugs scored at the bottom of our list and were also ranked much higher according to both degree and eigenvector centrality (fifth and 15th, respectively). Another similar observation was also noticed for a drug known as gadolinium where it was ranked fourth and third according to degree and eigenvector centrality, respectively, while CovidX suppressed its rank to the 21st position in the list. Figure 6 depicts the ranking comparison between CovidX and the eigenvector network centrality. The figure shows a slight overlap in the ranking, as in the case of tilorone dihydrochloride, which was scored in the 10th position of each measure. We also observed that an overlap in the ranking of the top 10, where chloroquine is in the third position versus the first position in degree of centrality. In addition, hydroxychloroquine is ranked as the top drug in CovidX, while it is ranked in the second position according to the eigenvector centrality.

Although studying the biomedical literature stored in PubMed has clearly paved the way to highlight the centrality of drugs and hence their significance to treat COVID-19, we also believe the role of the clinical trial factor, as an external factor, has transformed the ranking and provided real-world insights that the CovidX algorithm has rightfully used. Another significant aspect of incorporating the clinical trials factor is that it can be frequently updated to produce new ranks to the drugs as new clinical trials are launched.

It is important to note that the ranking algorithm we provide here is only a first step on the path of identifying drugs that can be repurposed. However, some of the findings of this paper have already elaborated on the importance of adjuvant therapy (more than one drug to work together) to treat the symptoms. Some drugs may focus on enhancing the immune system, another may enhance the lung function, and another could work against the virus. To achieve these therapies, further clustering analysis must be performed against the antiviral drugs listed in this paper as a starting point. Based on the outcome of this future study, another ranking mechanism might be needed to identify the most promising adjuvants.

In conclusion, this research presents a network analysis method to explore the SARS-CoV-2 literature to provide a comprehensive map of the drug mentions and their co-occurring biological features (eg, gene, protein, chemical compounds, cell). Such a knowledge by itself is significant and may enable more advanced science to identify not only a drug but also a biologic therapy or a vaccine. We have taken this research further by incorporating real-world evidence from clinical trials, and we made recommendations that are subject to change given new evidence. In the future, we hope to expand our network to look beyond publications that are not only concerned with SARS-CoV-2. We also hope to study other real-world evidence factors that may contribute to the future version of the CovidX algorithm.

### Limitations

The results of this research were strictly derived from the basis of existing research embedded in publications available in the PubMed archive.

### Conclusions

Given how dynamic the rapid development of this pandemic is, we conclude that our ranking here, though promising, is temporary and contingent upon future evidence. We also believe that it could be the case that a few drugs will be used as an adjuvant treatment as opposed to a single drug. Based on new evidence from the literature and clinical trials, the ranking will be a step toward a full treatment. Future efforts will invest in clustering the ranked drugs in an attempt to predict how adjuvant therapy may contribute to the full treatment of the disease.

## Appendix

Multimedia Appendix 1 Study input files, study intermediate output file, study results, and source code in Jupyter Notebooks.

## Abbreviations

ACE2: angiotensin-converting enzyme 2
ANG II: angiotensin II
ARDS: acute respiratory distress syndrome
CD: cluster of differences
COVID-19: coronavirus disease
CT: computed tomography
DIC: disseminated intravascular coagulation
IL: interleukin
MERS-CoV: Middle East respiratory syndrome–related coronavirus
RA: rheumatoid arthritis
RAAS: renin-angiotensin-aldosterone system
RdRp: RNA-dependent RNA polymerase
SARS: severe acute respiratory syndrome
SARS-CoV: severe acute respiratory syndrome–related coronavirus
SARS-CoV-1: severe acute respiratory syndrome coronavirus
SARS-CoV-2: severe acute respiratory syndrome coronavirus 2
SLE: systemic lupus erythematosus
Th: T helper
VKA: vitamin K antagonists
