# Supplementary material for: The Anatomy of the SARS-CoV-2 Biomedical Literature: Introducing the CovidX Network Algorithm for Drug Repurposing Recommendation
Source: J Med Internet Res. 2020 Aug 20;22(8):e21169. doi: 10.2196/21169 (PMC7474417; doi:10.2196/21169)
Supplement: Multimedia Appendix 1 [file jmir_v22i8e21169_app1.zip › input/UniProt_programmatically_py3.pdf]

UniProt_programmatically_py3

June 19, 2017

1 UniProt, programmatically

1.1 Part of the series: EMBL-EBI, programmatically: take a REST from manual searches

This notebook is intended to provide executable examples to go with the webinar. Code was tested in June 2017 against UniProt release 2017_06. Python version was 3.5.1

1.2 What is UniProt?

The UniProt Consortium provides many protein-centric resources at the center of which is the UniProt Knowledgebase (UniProtKB). The UniProtKB is what most people refer to when they say 'UniProt'. The UniProtKB sits atop and next to other resources provided by UniProt, like the UniProt Archive (UniParc), the UniProt Refernce Clusters (UniRef), Proteomes and various supporting datasets like Keywords, Literature citations, Diseases etc.

All of these resources can be explored and queried using the website www.uniprot.org. All queries run against resources are reflected in the URL, thus RESTful and open to programmatic access. Playing with the query facilities on the webiste is also a great way of learning about query fields and parameters for individual resources.

In addition, (fairly) recently added (often large scale) datasets are exposed only for programmatic access via a different base URL www.ebi.ac.uk/proteins/api or the Proteins API for short.

1.3 RESTful endpoints exposed on www.uniprot.org

Here is a list of all the RESTful endpoints exposed via the base URL http://www.uniprot.org:

- UniProtKB: /uniprot/ [GET]
- UniRef: /uniref/ [GET]
- UniParc: /uniparc/ [GET]
- Literature: /citations/ [GET]
- Taxonomy: /taxonomy/ [GET]
- Proteomes: /proteomes/ [GET]
- Keywords: /keywords/ [GET]
- Subcellular locations: /locations/ [GET]
- Cross-references databases: /database/ [GET]
- Human disease: /diseases/ [GET]

In addition, there are a few tools which can also be used programmatically:

- ID mapping: /uploadlists/ [GET]
- Batch retrieval: /uploadlists/ [GET]

As the UniProtKB has the most sophisticated querying facilities those will be used in the following examples. ID mapping and batch retrieval will feature, too.

1.3.1 Where to find documentation

There are help pages UniProt website. The page detailing the query fields for UniProtKB are of particular relevance as is the page listing database codes for mappings.

1.3.2 Examples

We will use a few examples to demonstrate how to use the UniProt RESTful services. Obviously, Python is the language of choice here. Only the standard library and one additional third party package, requests, will be used.

```
In [30]: import requests
```

```
In [31]: requests.__version__
```

```
Out[31]: '2.12.4'
```

Define a few constants first:

```
In [32]: BASE = 'http://www.uniprot.org'
         KB_ENDPOINT = '/uniprot/'
         TOOL_ENDPOINT = '/uploadlists/'
```

1.3.3 Example 1: querying UniProtKB

Using the advanced query functionality on uniprot.org, we try searching for proteins from mouse that have 'polymerase alpha' in their name and are part of the reviewed dataset in UniProtKB (aka Swiss-Prot). The following search string does just that:

name:"polymerase alpha" AND taxonomy:mus AND reviewed:yes

This translates into the following URL (a few space added):

http://www.uniprot.org/uniprot/?query=name %3A%22polymerase +alpha%22+AND+ taxonomy%3Amus +AND+reviewed %3Ayes&sort=score

Making a tiny modification, the above URL can be plugged straight into a script for programmatic access: we replace the bit at the end 'sort=score' with 'format=list'. The latter retrieves results as a list of (UniProt) accessions.

```
In [33]: # URL string is split across three lines for better readbility
         fullURL = ('http://www.uniprot.org/uniprot/?'
                    'query=name%3A%22polymerase+alpha%22+AND+taxonomy%3Amus+AND+reviewed%3Ayes&'
                    'format=list')
```

In the above URL string, please note the base URL, www.uniprot.org, and the targetted REST endpoint, /uniprot/. In addition, the actual search query and the format for result retrieval are supplied in `key=value` form.

Next, we will use the requests library we loaded earlier to get the results. RESTful services use a small set of verbs for communication, one of which is GET, which requests provides as the function 'get'.

We provide `fullURL` as a parameter to `requests.get()` and store what's retrieved in a variable called `result`.

```
In [34]: result = requests.get(fullURL)
```

`result` is an object which contains more than just the data we got from UniProt. We can use it to check whether the query was successful (`result.ok`) at all and, if not, which HTTP status code the server returned.

```
In [35]: if result.ok:
             print(result.text)
         else:
             print('Something went wrong ', result.status_code)
```

```
P33609
P33611
Q61183
P25206
```

We can compare the result we got with what uniprot.org returns:

```
In [36]: %%HTML
         <iframe width='800' height='400' src='http://www.uniprot.org/uniprot/?query=name%3A%22polymera
```

```
<IPython.core.display.HTML object>
```

Response headers can be inspected As mentioned before, `result` is an object encapsulating the response the UniProt server sent back when we asked it for data. That that object contains more than just the actual UniProt data we already made use of by checking result.ok. There is even more in the response from the server, most of which is stored in something called the header.

Let's inspect the header in our `result` (it is a dictionary):

```
In [37]: for key, value in result.headers.items():
             print('{}: {}'.format(key, value))
```

```
Date: Mon, 19 Jun 2017 13:12:56 GMT
Server: Apache-Coyote/1.1
Vary: User-Agent
X-Hosted-By: European Bioinformatics Institute
Content-Type: text/plain;charset=UTF-8
X-UniProt-Release: 2017_06
Expires: Mon, 26 Jun 2017 13:12:57 GMT
X-Total-Results: 4
Access-Control-Allow-Origin: *
Last-Modified: Wed, 07 Jun 2017 00:00:00 GMT
Access-Control-Allow-Headers: origin, x-requested-with, content-type
Content-Length: 28
Keep-Alive: timeout=5, max=100
Connection: Keep-Alive
```

Any interesting bits? How about these?

'X-UniProt-Release': '2017_06' -> the current UniProt release!

'X-Total-Results': '4' -> the number of returned results!

Despite our initial success in retrieving data from uniprot.org programmatically, the whole approach was not entirely satisfactory because we had to copy the full URL containing the query from uniprot.org in the first place. Would it not be nicer to provide the query string as we would write into the search box on uniprot.org? As a reminder, the query string was:

`name:"polymerase alpha" AND taxonomy:mus AND reviewed:yes`

We can do this!

All key=value parameters for the HTTP request can be provided to the `requests` module as a dictionary (which we call `payload`). The dict can then be passed to a dedicated parameter of the `requests.get()` function called `params`.

While we are at it, we will also construct our RESTful endpoint from the constans `BASE` and `KB_ENDPONIT` which we defined above.

```
In [38]: payload = {'query': 'name:"polymerase alpha" AND taxonomy:mus AND reviewed:yes',
                    'format': 'list'}
```

```
        result2 = requests.get(BASE + KB_ENDPOINT, params=payload)

        if result2.ok:
            print(result2.text)
        else:
            print('Something went wrong ', result.status_code)
```

```
P33609
P33611
Q61183
P25206
```

Need a diffent result format? Say, UniProt entries in plain text?
Just change the value for parameter `format` to `txt`:

```
In [39]: payload = {'query': 'name:"polymerase alpha" AND taxonomy:mus AND reviewed:yes',
                    'format': 'txt'}

        result2 = requests.get(BASE + KB_ENDPOINT, params=payload)

        if result2.ok:
            print(result2.text[:200])
        else:
            print('Something went wrong ', result.status_code)
```

```
ID   DPOLA_MOUSE             Reviewed;        1465 AA.
AC   P33609;
DT   01-FEB-1994, integrated into UniProtKB/Swiss-Prot.
DT   01-JUN-1994, sequence version 2.
DT   07-JUN-2017, entry version 149.
D
```

Available data formats When querying UniProtKB, several download formats are available.

- list: a list of accession numbers
- txt: UniProt entries in plain text format
- xml: UniProt entries in XML format
- tab: selected fields in tabular format

  This requires another parameter, `columns`, to specify which fields are of interest.
- fasta: sequences in FASTA format

  Unless used together with another parameter, `include=yes`, this only retrieves the canonical sequence, i.e., one sequence per entry.
- gff: annotated sequence features in GFF format
- rdf: UniProt entries in RDF format

In general, available formats vary with the RESTful service that is used.

Using the tabular format Here is a look at the tabular format:

```
In [40]: payload = {'query': 'name:"polymerase alpha" AND taxonomy:mus AND reviewed:yes',
                    'format': 'tab',
                    'columns': 'id,entry_name,reviewed,protein_names,organism,ec,keywords',
                    #'columns': ','.join(['id','entry_name','reviewed','protein_names',
                    #                     'organism','ec','keywords']),
                   }
         result = requests.get(BASE + KB_ENDPOINT, params=payload)
         if result.ok:
             print(result.text[:200])
         else:
             print('Something went wrong: ', result.status_code)
```

```
Entry        Entry name        Status        Protein names        Organism        EC number        Keyw
P33609        DPOLA_MOUSE        reviewed        DNA polymerase alpha catalytic subunit (EC 2.7.7.7) (DN
```

Downloading FASTA-formatted sequences And here an example retrieving sequences including isoforms:

```
In [41]: payload = {'query': 'name:"polymerase alpha" AND taxonomy:mus AND reviewed:yes',
                    'format': 'fasta',
                    # comment the following line to exclude isoforms
                    'include': 'yes',
                   }
         result = requests.get(BASE + KB_ENDPOINT, params=payload)
         if result.ok:
             print(result.text[:200])
         else:
             print('Something went wrong: ', result.status_code)
```

```
>sp|P33609|DPOLA_MOUSE DNA polymerase alpha catalytic subunit OS=Mus musculus GN=Pola1 PE=1 SV=2
MAPMHEEDCKLEASAVSDSGSFAASRARREKKSKKGRQEALERLKKAKAGEKYKYEVEDL
TSVYEEVDEEQYSKLVQARQDDDWIVDDDGIGYVEDGREIFD
```

What happens when a nonsensical query is launched? In the examples above, an `if - else` statement was used to react to the server response not being OK. If all is well, the server returns code 200 which we test in `if result.ok`. If the server encountered an error, a different code would be returned and handled in the `else` statement. Note, however, that a nonsensical query returning zero hits would still be OK from the server's point of view - `result.text` would be empty though.

We can demonstrate this. Re-using the code downloading FASTA-formatted sequences from above, we introduce a deliberate typo, plymerase, in the query so that we are unlikely to get any results (unless, say, a TrEMBL sequence has the exact same typo in its name). An extra `print` statement provides feedback:

```
In [42]: baseURL = 'http://www.uniprot.org/uniprot/'
         payload = {'query': 'name:"plymerase alpha" AND taxonomy:mus AND reviewed:yes',
                    'format': 'fasta',
                    # comment the following line to exclude isoforms
                    'include': 'yes',
                   }
         result = requests.get(baseURL, params=payload)
         if result.ok:
             print('Server response was OK, code {}'.format(result.status_code))
             print(result.text[:200])
         else:
             print('Something went wrong: ', result.status_code)
```

```
Server response was OK, code 200
```

1.3.4 Example 2: Mapping database identifiers

As mentioned earlier, there is a RESTful service for mapping database identifiers. It is exposed via the following base URL: `http://www.uniprot.org/uploadlists/`.

What does it allow us to do?

- map a UniProt accession to another database identifier
- map other database identifiers to a UniProt accession
- retrieve many UniProt entries in one go (by mapping from UniProt to UniProt)
- ~~map some DB identifier to another DB identifier directly~~
- map identifiers from DB_1 to DB_2 via UniProt accessions

As an example, we will map a set of PDB identifiers to ENSEMBL ones via UniProt accessions. In order to do this we have to step up our Python a tiny bit and write a function. This will allow us to re-use code and chain two calls to the mapping service.

```
In [43]: def map_retrieve(ids2map, source_fmt='ACC+ID',
                          target_fmt='ACC', output_fmt='list'):
             '''Map database identifiers from/to UniProt accessions.

             The mapping is achieved using the RESTful mapping service provided by
             UniProt. While a great many identifiers can be mapped the documentation
             has to be consulted to check which options there are and what the database
             codes are. Mapping UniProt to UniProt effectlvely allows batch retrieval
             of entries.

             Args:
                 ids2map (list or string): identifiers to be mapped
                 source_fmt (str, optional): format of identifiers to be mapped.
                     Defaults to ACC+ID, which are UniProt accessions or IDs.
                 target_fmt (str, optional): desired identifier format. Defaults
                     to ACC, which is UniProt accessions.
                 output_fmt (str, optional): return format of data. Defaults to list.

             Returns:
                 mapped identifiers (str)
             '''
             if hasattr(ids2map, 'pop'):
                 ids2map = ' '.join(ids2map)
             payload = {'from': source_fmt,
                        'to': target_fmt,
                        'format': output_fmt,
                        'query': ids2map,
                        }
             response = requests.get(BASE + TOOL_ENDPOINT, params=payload)
             if response.ok:
                 return response.text
             else:
                 response.raise_for_status()
```

The function name, `map_retrieve`, was chosen arbitrarily. The function takes a number of parameters as detailed in the docstring. The function returns data as a string; this could be done differently, of course.

Let's use it!

```
In [44]: pdb_ids = ['1YCS', '2AC0', '5UN8', '4S2Q']
         uniprot_acc = map_retrieve(pdb_ids, source_fmt='PDB_ID')
         print(uniprot_acc)
```

```
Q13625
O60502
P04637
Q04887
```

Now that we have UniProt accession corresponding to the PDB identifiers, we can use the former as input to yet another mapping call, this time retrieving ENSEMBL identifiers:

```
In [45]: # .strip().split() is used to transform the string of accessions
         # into a Python list.
         ensembl = map_retrieve(uniprot_acc.strip().split(), target_fmt='ENSEMBL_ID')
         print(ensembl)
```

```
ENSG00000141510
ENSG00000143514
ENSG00000198408
ENSMUSG00000000567
```

And we are done with the mapping.

1.4 RESTful endpoints exposed via the Proteins API

There are many RESTful endpoints exposed via the base URL www.ebi.ac.uk/proteins/api. The page also provides the documentation of the various services and it does that in an interactive manner. All the induvidual RESTful services can be explored and played with while reading the documentation. This makes learning to use them quite easy and fewer examples are needed.

Here is a view of the page:

```
In [46]: %%HTML
         <iframe width='800' height='400' src='http://www.ebi.ac.uk/proteins/api/doc'>
```

```
<IPython.core.display.HTML object>
```

1.4.1 Example 3: Searching for proteomics peptides

Just one example here to illustrate how one of the proteomics endpoints can be used to map a set of peptides like they could be generated by a proteomics experiment to UniProt entries. The endpoint we are talking about here is `http://www.ebi.ac.uk/proteins/api/proteomics`. Find this in the page above, set `Response Content Type` to json, paste these two peptides - rvlslgr,ldeafefvk - into the field for parameter `peptide` and then click the button `Try it out!`.

Various bits will be displayed then - the `Response Body`, a `Request URL` and also some `Request sample Code`. For the latter, there is a tab called `Python`. I copied the code from it below.

```
In [47]: # This we already imported earlier
         #import requests
         import sys

         # wrapped the string for better readability
         requestURL = ("https://www.ebi.ac.uk/proteins/api/proteomics"
                       "?offset=0&size=100&peptide=rvlslgr%2Cldeafefvk")
```

```
r = requests.get(requestURL, headers={ "Accept" : "application/json"})

if not r.ok:
  r.raise_for_status()
  sys.exit()

responseBody = r.text[:50]
print(responseBody)
```

```
[{"accession":"P28562","entryName":"DUS1_HUMAN","s
```

In order to make the code reusable we could write a function etc. For now, we will just modify it so that the value for parameter `peptide` can be provided in a nicer way. We also provide the base URL and the endpoint as constant to highlight the modular structure.

```
In [48]: PROTEINS_BASE = 'https://www.ebi.ac.uk/proteins/api'
         PROTEOMICS_ENDPOINT = '/proteomics'

         payload = {'peptide': 'rvlslgr,ldeafefvk'}
         # If starting from a Python list:
         #payload = {'peptide': ','.join(['rvlslgr', 'ldeafefvk'])}

         r = requests.get(PROTEINS_BASE + PROTEOMICS_ENDPOINT, params=payload, headers={ "Accept" : "ap

         if not r.ok:
           r.raise_for_status()
           sys.exit()

         responseBody = r.text[:50]
         print(responseBody)
```

```
[{"accession":"P28562","entryName":"DUS1_HUMAN","s
```
