# Supplementary figures and images for: The Anatomy of the SARS-CoV-2 Biomedical Literature: Introducing the CovidX Network Algorithm for Drug Repurposing Recommendation
Source: J Med Internet Res. 2020 Aug 20;22(8):e21169. doi: 10.2196/21169 (PMC7474417; doi:10.2196/21169)

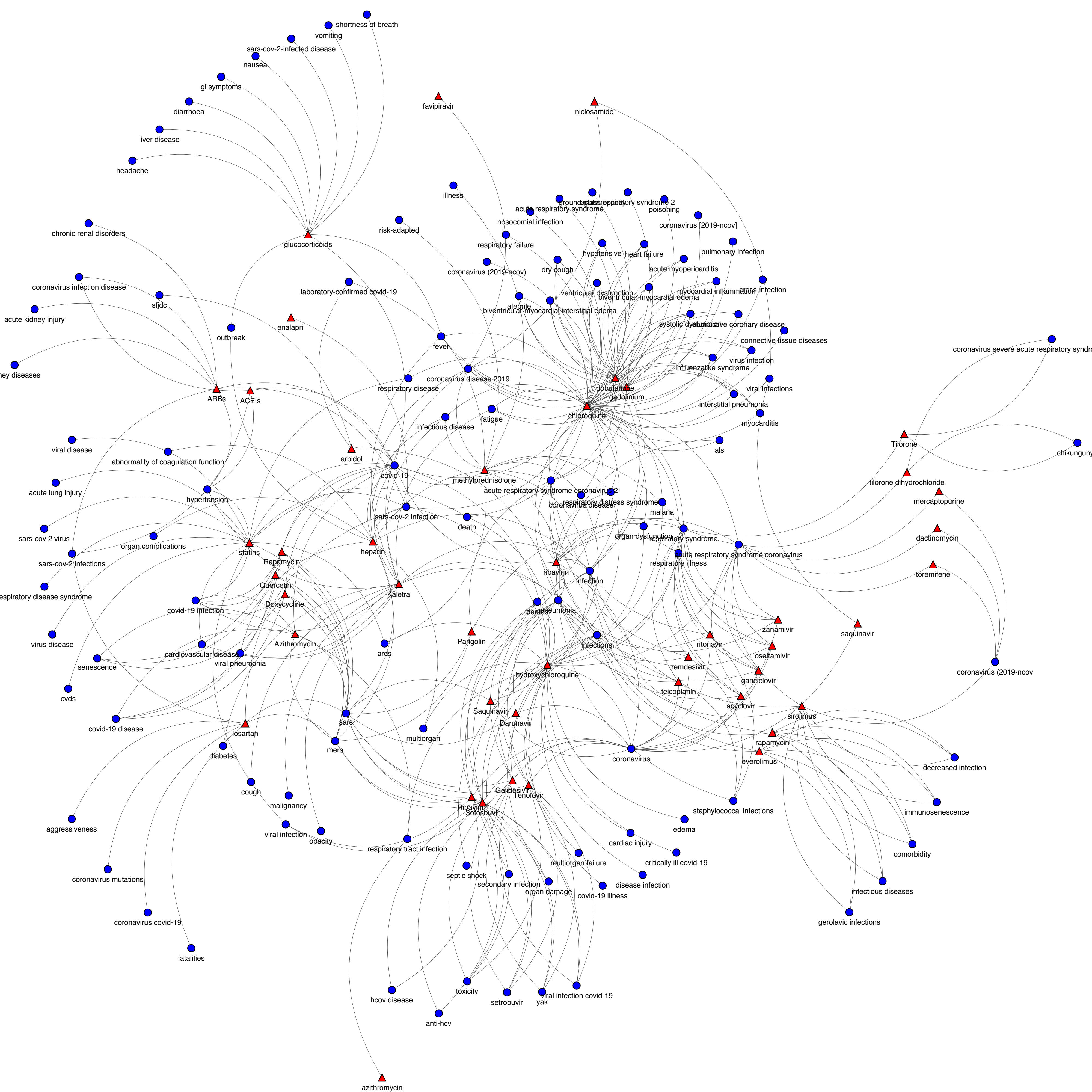

Supplement: Multimedia Appendix 1 [file jmir_v22i8e21169_app1.zip › results/drug-disease-2.pdf]
